# Saffron reduces the liver fibrosis in mice by inhibiting the JAK/STAT3 pathway

**DOI:** 10.1590/acb385823

**Published:** 2023-12-01

**Authors:** Lijuan Huang, Yan Han, Zhi Wang, Qiao Qiu, Sichen Yue, Qingmin Zhou, Wei Su, Jianhui Yan

**Affiliations:** 1 Xiangnan University – College of Nursing – Chenzhou, Hunan Province – People’s Republic of China.; 2 Xiangnan University -Affiliated Hospital – Chenzhou, Hunan Province – People’s Republic of China.

**Keywords:** Liver Cirrhosis, Crocus, Inflammation

## Abstract

**Purpose:**

Chronic inflammation in the liver is a key trigger for liver injury and fibrosis in various liver diseases. Given the anti-inflammatory and antioxidant effects of Saffron, this study aimed to investigate the pharmacological effects of Saffron on hepatic inflammation and fibrosis.

**Methods:**

The mice model of hepatic fibrosis was constructed using CCl_4_, and Saffron was administered at low (10 mg/kg) and high (20 mg/kg) doses by gavage. Then, the changes in liver function, liver inflammation and fibrosis markers were evaluated. The effects and mechanisms of Saffron on hepatic stellate cells were further investigated in in-vitro experiments.

**Results:**

Saffron improved liver function, reduced liver inflammation and attenuated liver fibrosis in a dose-dependent manner in hepatic fibrosis mice. Furthermore, Western blotting showed that Saffron significantly inhibited JAK/STAT3 phosphorylation in fibrotic livers.

**Conclusions:**

Saffron can attenuate liver fibrosis by inhibiting the JAK/STAT3 pathway and the activation of hepatic stellate cell, providing a theoretical basis for the development of new anti-fibrotic drugs.

## Introduction

Liver fibrosis is a chronic liver injury process caused by a variety of triggers such as viral infection, drug-induced injury, alcohol consumption and metabolic diseases. With the development of liver fibrosis, extracellular matrix protein deposition, liver fibrosis develops into cirrhosis, and even increases the risk of cancer^1^. There is no effective treatment for liver fibrosis recently. Therefore, it is an urgent need to find effective drugs to prevent or treat liver fibrosis.

During chronic liver injury caused by different etiologies, liver inflammation is a major contributor to liver pathology^2^. When the liver is injured, the production of inflammatory factors and reactive oxygen species (ROS) increases, and these intermediates can induce pro-fibrosis mechanisms^3^. The activation of hepatic stellate cells (HSCs) is considered to be a key event in the development of liver fibrosis^4^. Inflammatory factors play an important role in the activation of HSCs and further mediate the differentiation, proliferation, and collagen production of these cells, which cause excessive accumulation of extracellular matrix and lead to the development of liver fibrosis/cirrhosis^5^. In addition, ROS can trigger lipid peroxidation of biofilms and alter the membrane structure of hepatocytes and sub-organelles, resulting in a large number of inflammatory mediators and cytokines, which further aggravate liver damage^6^. Therefore, inhibiting liver inflammation and oxidative stress is an effective strategy to combat liver fibrosis.

Saffron is a traditional spice and herb that has been used as a food seasoning. In traditional medicine, it is used to treat coughs, colds, insomnia, spasms, asthma and bronchospasm, liver disease, pain, and epilepsy^7^^,^ 8. Recent studies have shown that saffron also has important functions in antioxidant, anti-inflammatory, and anti-apoptotic activity, which mainly due to its main active ingredient^9^^,^ 10. Chu et al.^11^ reported that saffron protected the doxorubicin-induced cardiotoxicity by inhibiting oxidative stress, inflammation, apoptosis, and correcting cardiomyocyte calcium homeostasis and mitochondrial damage. Oral saffron (100 mg/kg) for 21 days has been reported to prevent spatial memory deficits and oxidative stress in rats caused by streptozotocin and plays an important role in the prevention and control of Alzheimer’s disease^12^. However, the role of saffron on liver fibrosis has been rarely reported.

There is growing evidence that the signal transcriptional activator 3 (STAT3) is closely related to a range of inflammatory mediators and functions as key signal transduction molecules in many inflammatory diseases. Once STAT3 is activated and phosphorylated, it can be transferred to the nucleus and to regulate gene expression, thereby regulating cell growth, apoptosis, survival, and migration^13^. Blocking interleukin (IL)-6-mediated STAT3 signal activation has been shown to be beneficial in the treatment of colitis in mice^14^. IL-22 activates STAT3 by initiating the JAK and TYK kinases associated with IL-22 receptors, which in turn mediate host defense, metabolic reprogramming, intestinal inflammation, and carcinogenesis^15^.

In this study, we explored the anti-fibrosis efficacy of Saffron and its mechanism. Our results suggest that Saffron inhibits the activation of HSCs by JAK/STAT3 signaling pathway, thereby alleviating liver fibrosis in mice, laying a theoretical foundation for finding new therapeutic methods and drugs to improve the clinical outcomes of patients with liver fibrosis in the future.

## Methods

### Animal experiments

Male C57BL/6 mice at 8 weeks old were purchased from the Institute of Laboratory Animal Science, Chinese Academy of Medical Science (Beijing, China). Mice were housed under controlled conditions of 20°C (± 2°C) and 12-h light/dark cycles of 40–50% relative humidity, with free access to water and standard food. One week after adaptive rearing, mice were intraperitoneal injection with CCl_4_ (1 mL/kg of body weight dissolved in corn oil, fin al concentration of 20%) twice a week for six weeks, to construct liver fibrosis models. The control group received an equal amount of corn oil injection. After six weeks, mice were randomly divided into four groups (n = 6/group). The control and CCl_4_ groups were given normal saline daily by gavage, and 10- and 20-mg/kg Saffron were given in the low-dose and high-dose treatment groups per day for four weeks. All mice were sacrificed 24 h after the last treatment, and serum and liver samples were collected for subsequent experiments.

### Biochemical parameters

The contents of aspartate transaminase (AST) and alanine transaminase (ALT) in mouse serum were tested by using standard autoanalyzer methods on Chemray 240 automatic biochemistry analyzer (Rayto, United States of America).

### Hematoxylin-eosin and Sirius red staining

Liver tissue was fixed in 4% paraformaldehyde, embedded in paraffin, sectioned, and stained with hematoxylin and eosin (H&E) and Sirius red according to a standard procedure. ImageJ software was used to analyze the Sirius red–positive areas. The percentage of the fibrotic area was calculated in five randomly selected fields per slide.

### Real-time quantitative polymerase chain reaction

The total RNA was extracted from liver tissues using Trizol reagent. Concentration and purity of RNA were measured using a NanoDrop 1000 spectrophotometer, and total RNA was reverse transcribed to cDNA using the cDNA reverse transcription kit (Takara Bio, China) according to the manufacturer’s protocol. Follow the real-time polymerase chain reaction (RT-PCR) kit instructions (Takara Bio, China) with glyceraldehyde 3-phosphate dehydrogenase (GAPDH) as the internal reference and three complex wells per sample. The primer sequences are as follows: IL-1β forward primer: 5’-TGCCACCTTTTGACAGTGATG-3’, reverse primer: 5’- ATGTGCTGCTGCGAGATTTG-3’;IL-6 forward primer: 5’- ACCAGAGGAAATTTTCAATAGGC-3’, reverse primer: 5’- TGATGCACTTGCAGAAAACA-3’;IL-10 forward primer: 5’-TTCTTTCAACAAAGGACCAGC-3’, reverse primer: 5’-GCAACCCAAGTAACCCTTAAAG-3’; tumor necrosis factor-α (TNF-α) forward primer: 5’-GCATGATCCGAGATGTGGAACTGG-3’, reverse primer: 5’-CGCCACGAGCAGGAATGAGAAG-3’; GAPDH forward primer: 5’-AGGTCGGTGTGAACGGATTTG-3’, reverse primer: 5’-CGCCACGAGCAGGAATGAGAAG-3’. The data were processed using a relative expression of 2^-ΔΔCt^.

### Cell culture

Human hepatic stellate cell LX-2 was purchased from the Typical Culture Preservation Center of the Chinese Academy of Sciences (Shanghai, China). LX-2 cells were cultured with high-sugar Eagle’s minimal essential medium (DMEM) (HyClone, United States of America) containing 10% fetal bovine serum, 1% penicillin-streptomycin, placed in a 37 °C, 5% carbon dioxide incubator, and the culture solution was changed every two days. LX-2 was activated by transforming growth factor-β1 (TGF-β1) (5 ng/mL) for 24 h. Colivelin (a STAT3 activator) was used to activate the JAK/STAT3 signaling.

### Cell viability assay

Cell viability assays were performed using MTT (Sigma, United States of America) colorimetry. LX-2 cells were seeded in 96-well plates (1 × 104 cells/well) with three rewells per group. After 24 h of incubation, a series of concentrations of Saffron (0, 5, 10, 20, 40, 100 μM) were added, and, after continuing the culture for 24 and 48 h, 10 μL of MTT reagent was added per well, placed in an incubator for 1 h and then determined the absorbance value at the wavelength of 490 nm using a microplate reader. The experiment was repeated for three times.

### Western blotting

Proteins in mouse liver tissues and Saffron-treated LX-2 cells were extracted with RIPA lysis buffer, and protein concentrations were detected with the BCA protein concentration determination kit (Biyuntian, Shanghai). SDS-PAGE electrophoresis was performed, the isolated proteins were transferred onto a polyvinylidene difluoride (PVDF) membrane, and they were blocked with 5% skimmed milk powder for 1 h. The film was washed three times with TBST, and the corresponding primary antibodies were added: Collagen I (Abcam, ab138492), α-SMA (Sigma, A5228), TGF-β1 (Abcam, ab215715), JAK (Cell Signaling, #3230), p-JAK (Cell Signaling, #3771), STAT3 (Cell Signaling, #9139), p-STAT3 (Cell Signaling, #9145), and GAPDH (Abcam, ab9485), besides being incubated overnight at 4°C. After the membrane being washed with TBST, horseradish peroxidase-labeled secondary antibodies were added, and the mixture was incubate for 1 h. The enhanced chemiluminescence substrate was chemiluminescent and colorated with ImageJ software for band analysis. The experiment was repeated by three times.

### Statistical analysis

The results were expressed as mean ± standard deviation, Statistical Package for the Social Sciences 20.0 software was used for data analysis, the comparison between the two groups was analyzed by the Student’s t-test, and the one-way analysis of variance was used for the comparison between multiple groups. The difference between P < 0.05 was statistically significant.

## Results

### Saffron alleviates liver fibrosis in mice

**Figure 1:**
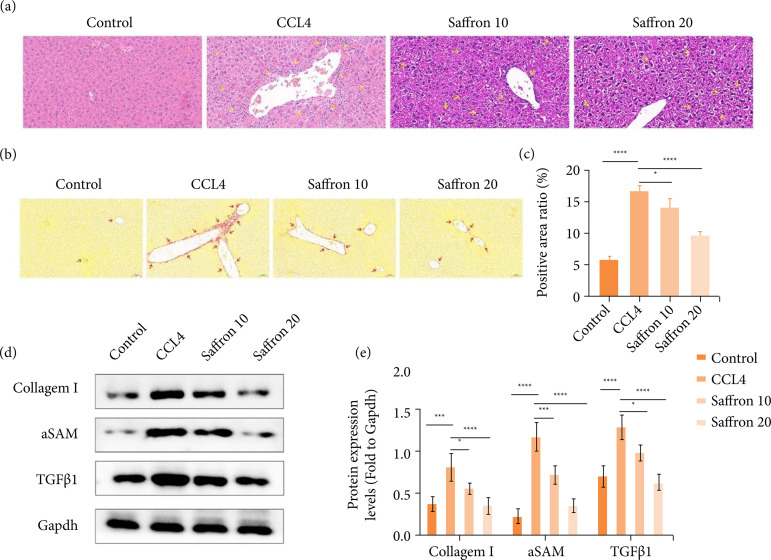
Saffron alleviates liver fibrosis in mice. **(a)** Representative hematoxylin and eosin staining, scale: 100 μM; **(b)** representative Sirius red staining, scale: 50 μM; **(c)** quantification of Sirius’ red-positive regions; (**d-e**) determination of expression levels of fibrosis marker proteins collagen I, α-SMA and TGF-β1 in liver tissues by Western blotting.

H&E staining and Sirius red staining were used to assess the therapeutic effect of saffron on liver fibrosis in mice. As shown in Figs. 1a–1c, the liver tissue of mice in CCl_4_ group exhibited increased hepatocyte degeneration and inflammatory cell infiltration and more fibrin deposition compared to the control group. Saffron treatment reduced hepatocellular degeneration and inflammatory cell infiltrates and the area of liver fibrosis in a dose-dependent manner.

We next measured the levels of fibrosis-related genes in liver tissue, including TGF-β1, α-SMA, and collagen I, all of which were lower in control group and significantly higher after CCl_4_ administration. Compared with the CCl_4_ group, the expression levels of these indicators were significantly reduced in the 10- and 20-mg/kg saffron treatment groups, and the reduction was greater in the 20 mg/kg saffron group (Figs. 1d-1e). These data suggest saffron was able to mitigate liver fibrosis in mice in a dose-dependent manner.

TGF: transforming growth factor; *p < 0.05; **p < 0.01; ***p < 0.001. Source: Elaborated by the authors.

### Saffron improves liver function and reduces liver inflammation in mice with liver fibrosis

**Figure 2:**
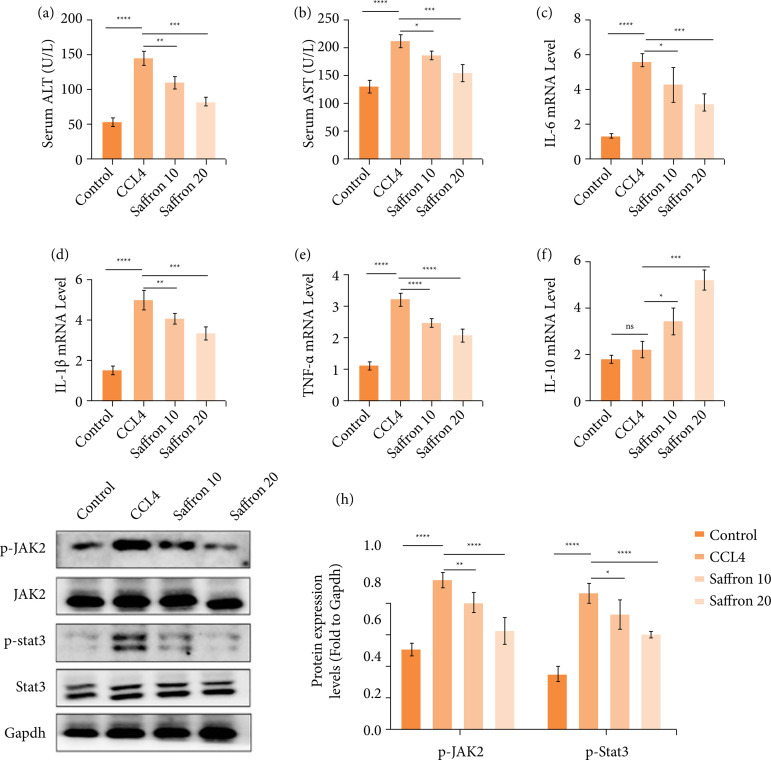
Saffron improves liver function in mice with liver fibrosis and reduces inflammation of liver tissue. (**a-b**) Biochemical analysis of the content of liver function indicators ALT and AST in the serum of mice; (**c–f**) real-time quantitative polymerase chain reaction detected the mRNA expression levels of inflammation-related factors IL-1β, IL-6, IL-10 and TNF-α in mouse liver tissues. (**g-h**) Phosphorylation of the JAK/STAT3 pathway in liver tissue was detected by Western blotting.

ALT: alanine transaminase; AST: aspartate transaminase; IL: interleukin; TGF: transforming growth factor; *p < 0.05; **p < 0.01; ***p < 0.001.Source: Elaborated by the authors.

We examined the effects of saffron on liver function and inflammation in liver fibrosis mice. Biochemical measurements showed that, compared with the control group, the serum AST and ALT levels in the CCl_4_ group were significantly increased, while these indicators were reduced in the 10- and 20-mg/kg saffron treatment groups, and the decrease was more pronounced in the 20-mg/kg saffron group (Figs. 2a-2b). To detect liver inflammatory status, we measured the mRNA expression levels of IL-1β, TNF-α, IL-6, and IL-10 in mice liver tissues by qRT-PCR. It showed that the expression levels of pro-inflammatory factors IL-1β, TNF-α and IL-6 in CCl_4_ group were significantly increased compared with control group. Compared to the CCl_4_ group, saffron treatment group reduced the mRNA levels of pro-inflammatory factors, while increased the expression of the anti-inflammatory factor IL-10 (Figs. 2c–2f). These results suggest that saffron is effective in improving liver function and reducing liver inflammation in mice with liver fibrosis.

We next investigated the mechanism by which saffron exerted its protective effect in CCl_4_-induced liver fibrosis. Given that JAK/STAT3 signaling plays an important regulatory role in diseases characterized by chronic inflammation and fibrosis^16^, we examined the status of the JAK/STAT3 pathway in each group of mice by Western blotting. The results showed that the phosphorylated of JAK and STAT3 in the CCl_4_ group were significantly increased compared with the control group, and saffron treatment inhibited the activation of JAK/STAT3, and this inhibition effect was more pronounced in the 20-mg/kg saffron group (Figs. 2g-2h). It is shown that saffron can alleviate liver fibrosis by inhibiting the JAK/STAT3 pathway.

### Saffron inhibits the activation of hepatic stellate cells

**Figure 3:**
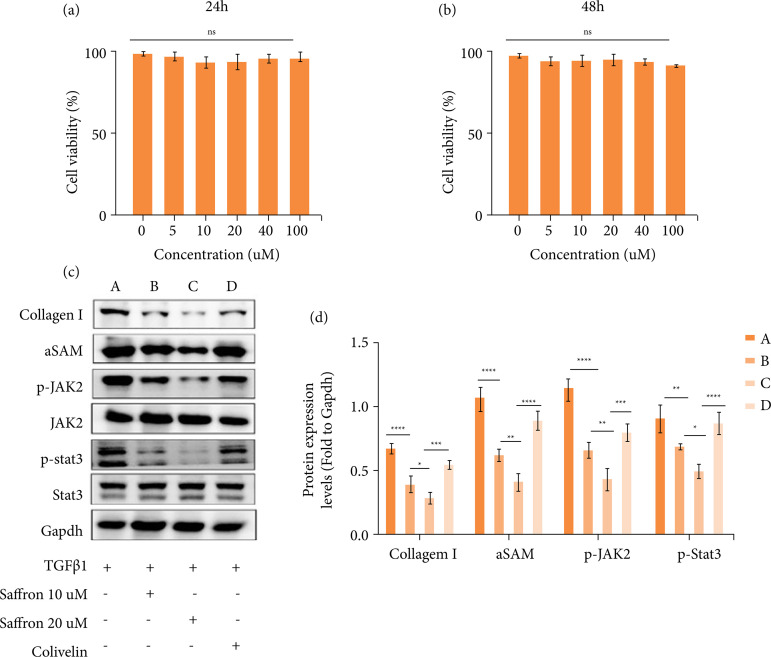
Saffron inhibits hepatic stellate cell activation. (**a-b**) Effect of MTT colorimetry on LX-2 cell viability by MTT colorimetry; (**c-d**) detection of expression of fibrosis markers collagen I and α-SMA by Western blotting, as well as phosphorylation of the JAK/STAT3 pathway.

GAPDH: glyceraldehyde 3-phosphate dehydrogenase; TGF: transforming growth factor; *p < 0.05; **p < 0.01; ***p < 0.001. Source:Elaborated by the authors.

We treated LX-2 with a series of concentrations of saffron and examined the effect of saffron on the cell viability of LX-2 after 24 and 48 h, and we found that there was no significant difference in cell viability between the intervention group and the control group (Figs. 3a-3b). Then, we examined the effect of 10- and 20-μM saffron on the activated LX-2. Western blotting showed that saffron significantly inhibited the expression of fibrosis markers α-SMA and collagen I, and 20-μM saffron decreased more than 10 μM (Figs. 3c-3d).

Next, we investigated the effect of Saffron on the JAK/STAT3 pathway in LX-2, and the results showed that saffron inhibited the expression of p-JAK and p-STAT3. To further explore the role of the JAK/STAT3 pathway in liver fibrosis, we added colivelin to the 20-μM saffron treatment group and found colivelin reversed the inhibitory effect of saffron on LX-2 (Figs. 3c-3d). These results indicated that saffron inhibited the activation of LX-2 in a dose-dependent manner and that this inhibitory effect was achieved by suppressing the JAK/STAT3 pathway.

## Discussion

Liver fibrosis is a complex pathological process that leads to the development of chronic liver diseases and cirrhosis, which not only leads to liver failure, but also to a series of complications^17^. There are currently no effective drugs for the treatment of liver fibrosis. Therefore, it is an urgent need to develop new therapies for inhibiting liver fibrosis.

Saffron has been used in the diet since ancient times. Its various components, such as saffron, zeaxanthin, lycopene, and separate alpha and beta-carotenes, have been reported to be effective in peptic ulcers, ulcerative colitis, colorectal cancer, and pancreatic diseases^18^^,^ 19. In this study, we treated liver fibrosis mice with saffron and found that saffron was effective in improving liver function, reducing inflammation, and inhibiting the expression of fibrosis-related proteins α-SMA, collagen I, and TGF-β1 in mice.

The persistent inflammation may trigger a fibrosis/cirrhosis response, leading to irreversible damage to liver cells and decreased liver function^20^. In the acute phase of CCl_4_-induced liver injury, hepatic inflammation triggers the activation and differentiation of HSCs from quiescent cells into myofibroblasts, acquiring proliferative, pro-inflammatory, and contractile properties, and contribute to the production of extracellular matrix, such as collagen, which leads to changes in liver structure and promotes the development of liver fibrosis^21^.

In this study, we examined the inflammation level of liver tissues in mice, the RT-qPCR results showed that saffron could significantly reduce the expression of pro-inflammatory factors IL-1β, TNF-α and IL-6, and increase the anti-inflammatory factor IL-10 expression, which suggesting that saffron may exert antifibrotic effects by reducing liver inflammation and inhibiting the activation of HSCs. TGF-β1 and α-SMA are considered the most prominent markers of fibrosis progress. Studies have confirmed that TGF-β1 contributes to HSCs activation, resulting in the production of pro-fibrosis cytokines and growth factors that promote the progression of fibrosis through autocrine and paracrine mechanisms^22^. Our study confirmed that CCl_4_ injection resulted in a significant increase in the expression levels of TGF-β1 and α-SMA, while saffron significantly eliminated the expression of TGF-β1 and α-SMA in liver tissues in a dose-dependent manner. Similar results were obtained in vitro; saffron inhibited the activation of HSCs and reduced the expression of fibrosis markers α-SMA and collagen I.

JAK, along with several STAT proteins, mediates signaling of a range of extracellular cytokines and affects a variety of cellular functions, of which STAT3 has been found to be a component of the IL-6-activated acute phase factor complex^23^. Several studies have reported an association between inflammation and STAT3 activation, and persistent or dysregulated STAT3 signaling can lead to many diseases, including chronic inflammation, fibrosis, and cancer, and STAT3 inhibitors are optimistic as treatments for these diseases, but caution is needed to avoid toxicity^16^. Oike et al.^24^ have suggested that STAT3 inhibitors can block joint inflammation and destruction in collagen-induced arthritis models^24^.

Therefore, we explored whether the JAK/STAT3 pathway is involved in the development of inflammation and fibrosis in the liver. Western blotting results showed that the phosphorylation of JAK and STAT3 in the liver tissues of saffron treatment groups were significantly inhibited, and the inhibition effect was more pronounced in the high-dose group. In-vitro experiments have also demonstrated that saffron inhibits the activation of HSCs by inhibiting the phosphorylation of JAK/STAT3. In addition, when we added the activator of STAT3, it was found that α-SMA and collagen I were partially reversed.

These results suggest that the activation of JAK/STAT3 can promote the development of liver fibrosis, and saffron can reduce liver inflammation by inhibiting the JAK/STAT3 pathway, thus improving liver function, and alleviating the progression of liver fibrosis. These results indicate that the activation of JAK/STAT3 can promote the development of liver fibrosis, and saffron can reduce liver inflammation, improve liver function, and mitigate the progression of liver fibrosis by inhibiting the JAK/STAT3 pathway.

## Conclusion

In conclusion, this study shows that saffron has an important role in alleviating liver fibrosis. Further studies confirmed that saffron mainly reduced liver inflammation and inhibited HSCs activation by inhibiting the JAK/STAT3 pathway, thereby reducing liver fibrosis. This finding may provide new strategies for the treatment of liver fibrosis.

## Data Availability

The datasets used and/or analyzed during the current study are available from the corresponding author on reasonable request.
